# Fas-Fas Ligand Interplay in the Periphery of Salivary Gland Carcinomas as a New Checkpoint Predictor for Disease Severity and Immunotherapy Response

**DOI:** 10.3390/biomedicines9040402

**Published:** 2021-04-08

**Authors:** Zuzana Strizova, Martin Kuchar, Linda Capkova, Martin Komarc, Jiri Skrivan, Jirina Bartunkova, Jan Plzak, Daniel Smrz

**Affiliations:** 1 Department of Immunology, Second Faculty of Medicine, University Hospital Motol, Charles University, 15006 Prague, Czech Republic; zuzana.strizova@fnmotol.cz (Z.S.); jirina.bartunkova@lfmotol.cuni.cz (J.B.); 2 Department of Otorhinolaryngology and Head and Neck Surgery, First Faculty of Medicine, University Hospital Motol, Charles University, 15006 Prague, Czech Republic; martin.kuchar@fnmotol.cz (M.K.); jan.plzak@fnmotol.cz (J.P.); 3 Department of Pathology and Molecular Medicine, Second Faculty of Medicine, Charles University, 15006 Prague, Czech Republic; linda.capkova@fnmotol.cz; 4 Department of Methodology, Faculty of Physical Education and Sport, Charles University, 15006 Prague, Czech Republic; komarc@volny.cz; 5 Department of Otorhinolaryngology, Second Faculty of Medicine, University Hospital Motol, Charles University, 15006 Prague, Czech Republic; jiri.skrivan@fnmotol.cz

**Keywords:** Fas, FasL, salivary gland carcinoma, tumor center, tumor periphery

## Abstract

Salivary gland carcinomas (SGCs) are extremely morphologically heterogeneous, and treatment options for this disease are limited. Immunotherapy with immune checkpoint inhibitors (ICIs) represents a revolutionary treatment approach. However, SGCs remain largely resistant to this therapy. An increasing body of evidence suggests that resistance to ICI therapy is modulated by the Fas (CD95)–Fas ligand (FasL, CD178) interplay between tumor cells and immune cells. In this study, we examined the Fas–FasL interplay between tumor cells and tumor-infiltrating immune cells (TIICs) in the center and periphery of SGCs from 62 patients. We found that the Fas-expressing tumor cells accumulated in the center of SGC tumors with increasing tumor stage. Furthermore, this accumulation occurred regardless of the presence of TIICs expressing high levels of FasL. On the contrary, a loss of Fas-expressing TIICs with increasing tumor stage was found in the tumor periphery, whereas FasL expression in tumor cells in the tumor periphery correlated with tumor stage. These data suggest that SGC cells are resistant to FasL-induced apoptosis by TIICs but could utilize FasL to eliminate these cells in high-stage tumors to provide resistance to immunotherapy.

## 1. Introduction

Primary tumors of the salivary glands are rare and broadly heterogeneous malignancies of the head and neck region [1]. Less than 5% of all cancers of the head and neck are salivary gland carcinomas (SGCs). SGCs include the most diverse cancer types in humans, with more than 20 histological subtypes [1,2]. The treatment option for resectable SGCs is surgery [2]. Surgery, however, requires a distinctive approach because of the extreme morphological heterogeneity of SGCs [3]. Despite this approach, a large number of SGCs have locoregional recurrence [4]. This recurrence is dependent on the tumor’s histological subtype, grade, and primary tumor stage and is reported in 15–80% of patients within 5 years of surgery [4]. In unresectable SGCs, the treatment options are chemotherapy or targeted therapy. Due to the unavailability of other treatment options, these two are frequently utilized, but they are mostly ineffective [2,4,5]. The 5-year survival of individuals with high-grade and recurrent SGCs is less than 35% [4].

A currently expanding treatment option is immunotherapy, which, in many cancer types, has already become the first-line treatment option [6,7,8]. The response rates to immunotherapy, however, vary among cancer types and, importantly, even among tumor cohorts of the same cancer type [9]. Therefore, the major challenge of immunotherapy is to not only predict immunotherapy responders [9] but also to identify the molecular mechanisms that could be targeted to overcome resistance to immunotherapy [10,11,12]. To this end, it is important to understand the molecular interplay between tumor cells and immune cells under specific disease states in individual tumors. Current studies largely focus on CTLA-4 and PD-1/PD-L1 checkpoint inhibitors [13,14]. However, this therapy in SGCs has not shown better response rates than chemotherapy [5]. An increasing body of evidence, however, suggests that resistance to checkpoint inhibitor therapy is modulated by the Fas (CD95)–Fas ligand (FasL, CD178) interplay between tumor cells and immune cells [15,16,17]. The binding of FasL to the Fas receptor serves as an apoptosis-inducing mechanism that both CD8^+^ T cells and natural killer (NK) cells use to eliminate neoplastically transformed cells [18]. This mechanism, however, has also been successfully employed by tumor cells and tumor-infiltrating myeloid-derived suppressor cells (MDSCs) to counterattack tumor-infiltrating immune cells (TIICs) [15]. Whether the Fas–FasL interplay could be employed by SGCs as a mechanism of resistance to the immune system and/or play a role in resistance to checkpoint inhibitor therapy is unknown.

In this study, we investigated the expression of Fas and FasL in the tumor cells and TIICs of 62 SGC patients. The localization of Fas and FasL expression in these cells was stratified into two tumor compartments: the tumor center and tumor periphery. The obtained data were then correlated with the clinicopathological data of the patients to determine whether the expression and compartmental localization of these molecules in the investigated cells reflected the disease severity and indicated the mechanism of resistance of SGCs to the immune system and immunotherapy.

## 2. Materials and Methods

### 2.1. Patients

In this study, the tumors of 62 SGC patients were analyzed. All these patients underwent surgery between January 2013 and December 2018 and provided written consent to participate in the study. All experimental protocols were approved by the ethical standards of the institutional and/or national research committee—the Ethics Committee of the University Hospital Motol in Prague (EK-1394/20)—and performed in accordance with the 1964 Helsinki Declaration and its later amendments or comparable ethical standards. Individual tumors were scored based on tumor grade and stage. Tumors were assigned either to a low-grade (0) or a high-grade (1) group. Staging was carried out using the 8th edition of TNM Classification of Malignant Tumors (TNM) and the International Union Against Cancer (UICC)/American Joint Committee on Cancer (AJCC) staging system for head and neck cancer [19]. The presence of lymph node metastases was examined in each patient.

### 2.2. Immunohistochemistry

Formalin-fixed paraffin-embedded (FFPE) tissue samples were obtained from the patients, and the diagnosis was determined by a well-experienced pathologist. Each tissue sample and its staining were scored manually. The quality of the manual scoring results was assessed by the intrarater reliability test to avoid subjective bias. Slides of 3-µm thickness were stained using the following antibodies: anti-Fas (CD95) (cat. #MA5-32489; clone JJ0942, Thermo Scientific, Waltham, MA, USA) and anti-FasL (CD95L) (cat. #506-2714; Zytomed, Berlin, Germany). Fas and FasL expression in tumor and immune cells were analyzed separately and within the center and periphery of the tumor. The tumor periphery was defined as the tumor–host interface extending one high-power field (HPF, 400-fold magnification) from the tumor’s edge. The proportion of positive cells was based on the tumor proportion score (TPS), which measures the percentage of tumor cells/TIICs that have a significant positive membrane staining (TPS = (*n* of Fas or FasL-stained tumor cells)/(total *n* of viable tumor cells) × 100; (*n* of Fas or FasL stained TIICs)/(total *n* of viable TIICs) × 100; *n* = cell count). Only strong membranous staining (tumor cells) or strong membranous staining together with cytoplasmic staining (TIICs) were regarded as positive [20]. The positivity of antibody staining was evaluated according to a previously published scoring system based on the percentage of tumor area covered by positive-stained cells [21,22,23,24]. The scoring was as follows: score 0 (no stain), negative; score 1 (weak staining), 1–10%; score 2 (moderate staining), 10–49%; and score 3 (strong staining), above 50%.

### 2.3. Statistical Analysis

The indicated sample size (*n*) was used to calculate the means ± SEMs. Bivariate associations between the variables under study were assessed by Spearman’s rank-order correlation coefficient (*r*). Differences in paired measurements were evaluated by the Wilcoxon signed-rank test using a Monte Carlo resampling approach. *p* values were determined by the indicated test (* *p* < 0.05, ** *p* < 0.01, *** *p* < 0.001, and **** *p* < 0.0001). *p* < 0.05 was considered significant. For statistical analyses, SPSS Statistical Software version 25.0 (SPSS, Chicago, IL, USA, accessed on 8 January 2021) and GraphPad Prism 6 (GraphPad Software, La Jolla, CA, USA, accessed on 8 January 2021) were used. For the purpose of graphical presentation, GraphPad Prism 6, Microsoft Excel, and Raw Graphs (rawgraphs.io, accessed on 10 January 2021) were used.

## 3. Results

### 3.1. The Differences between the Expression of Fas and FasL in Tumor Cells and TIICs Are Clustered in the Tumor Center

In this study, we analyzed tumor samples from 62 SGC patients. The samples contained 13 distinct histological subtypes (Table 1) of different grades, stages, and associations with the presence or absence of lymph node (LN) metastases (Table 2).

**Figure 1:**
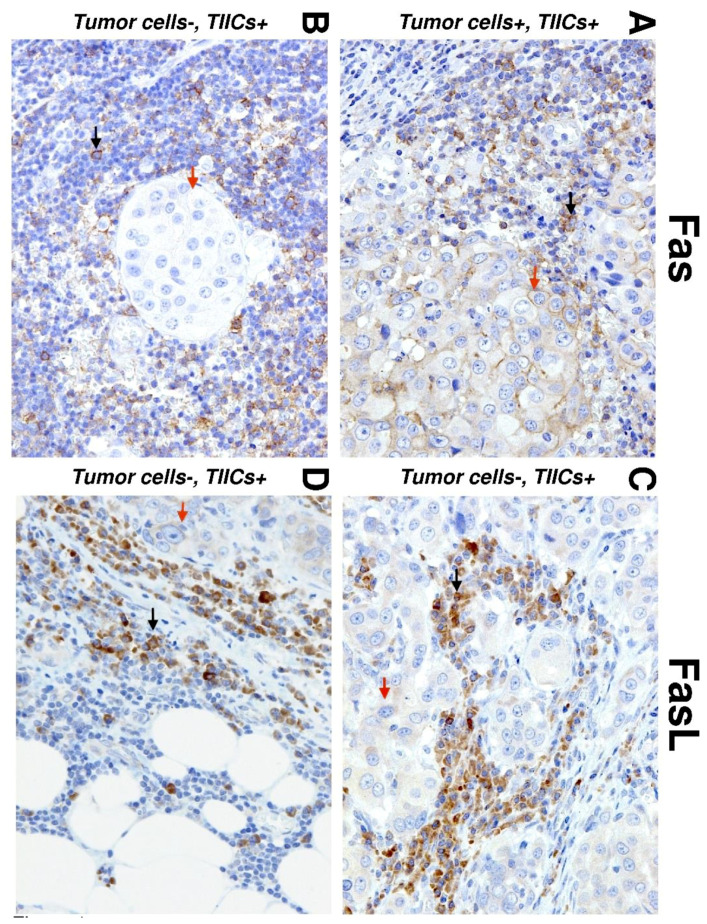
Immunohistochemistry (IHC) of Fas and Fas ligand (FasL) expression in salivary gland carcinoma (SGC) tissues. Representative images show Fas (left) and FasL (right) staining in tumor cells (red arrows) and tumor-infiltrating immune cells (TIICs) (black arrows) in the tumor periphery. (**A**) IHC shows positive Fas staining in TIICs and tumor cells (20×). (**B**) IHC shows negative Fas staining in tumor cells and positive Fas staining in TIICs (20×). (**C**) IHC shows positive FasL staining in tumor cells and TIICs (20×). (**D**) IHC shows negative FasL staining in tumor cells and positive FasL staining in TIICs (20×).

**Figure 2:**
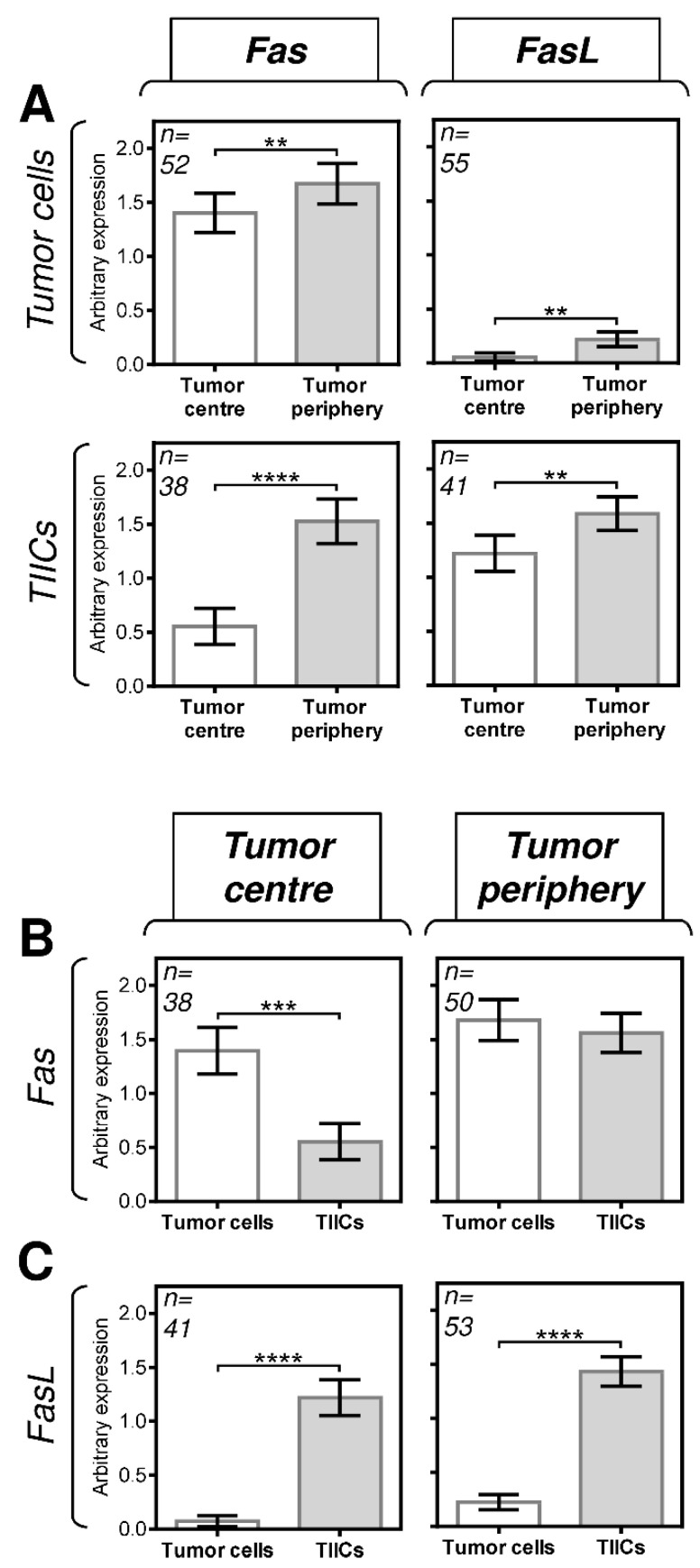
The expression of Fas and FasL in tumor cells and TIICs in the tumor periphery and center. (**A**) The expression (tumor proportion score (TPS)) of Fas and FasL in tumor cells and TIICs is higher in the tumor periphery than in the tumor center. The number of patients (*n*) evaluated is shown in the top left corner of each panel. (**B**) The expression of Fas in tumor cells is higher than that in TIICs in the tumor center but comparable to that in the tumor periphery. (**C**) The expression of FasL in tumor cells is lower than that in TIICs in both the tumor center and tumor periphery. In **A**–**C**, the expression analyses were performed according to the TPS scoring system described in the Materials and Methods section. The data are presented as the mean ± SEM of the TPS score calculated from the TPS scores of individual patients’ samples. The number of patients (*n*) evaluated is shown in the top left corner of each panel. The difference between groups was evaluated by the Mann–Whitney U test (*n* is shown in the top left corner of each panel, ** *p* < 0.01, *** *p* < 0.001, and **** *p* < 0.0001).

We first analyzed the expression of Fas and FasL in tumor cells and TIICs in the center and periphery of the tumors. The expression analyses were performed by immunohistochemistry using Fas- or FasL-specific antibodies (Figure 1). The expression levels in the tissue samples were analyzed according to the scoring system described in the Materials and Methods section. We found that both tumor cells and TIICs expressed more Fas and FasL in the tumor periphery than in the tumor center (Figure 2A). These data indicated that the Fas–FasL interplay between tumor cells and TIICs could be clustered in the periphery of tumors. Subsequent analyses, however, revealed that tumor cells and TIICs had comparable expression levels of Fas in the tumor periphery (Figure 2B, right panel) but different expression levels in the tumor center (Figure 2B, left panel). These data, therefore, suggested that the Fas-driven interplay between tumor cells and TIICs could also exist in the tumor center. On the other hand, these data suggested no such interplay for FasL because the differences in its expression between tumor cells and TIICs were comparable in both compartments, showing much higher FasL expression in TIICs than in tumor cells (Figure 2C).

### 3.2. Fas Expression in TIICs Negatively Correlates with Tumor Stage in the Tumor Periphery

The Fas–FasL interplay was previously found to be associated with disease severity and indicated possible mechanisms of tumor resistance to the immune system [15,16,25,26,27,28]. In the following series of analyses, we correlated the compartmental expression of Fas and FasL in tumor cells and TIICs with tumor grade, stage, and the presence/absence of lymph node (LN) metastases (Table 2). The tumors were stratified into two groups based on the grade: low-grade tumors (grade 0) or high-grade tumors (grade 1). The tumors were also stratified into two groups based on the presence or absence of metastases: absence of metastases (−) or presence of metastases (+). Finally, the tumors were stratified into four groups based on tumor stage: stage 1, 2, 3, or 4.

**Figure 3:**
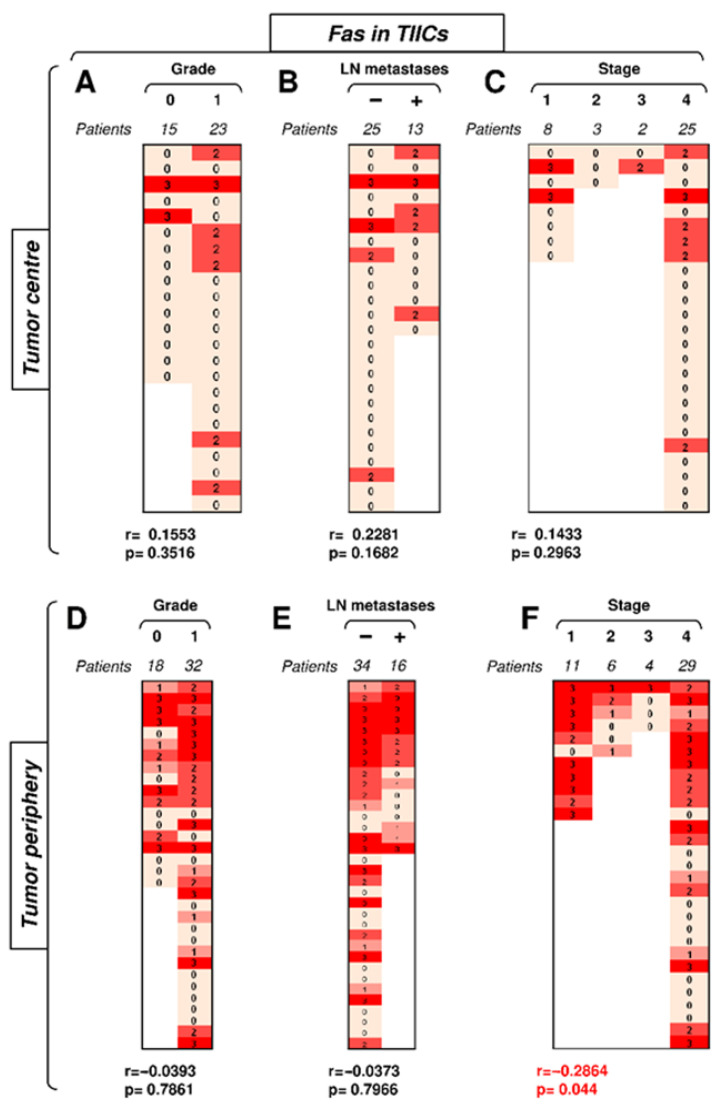
The expression of Fas in TIICs in the tumor periphery correlates with tumor stage. (**A**) The cohort of 38 SGC patients was stratified into 2 groups according to tumor grade (grade 0 and grade 1), and the Spearman correlation (*r*) according to the expression of Fas in TIICs in the tumor center was determined. The expression analyses were performed according to the scoring system described in the Materials and Methods section. The data are presented as a heat map with the scores, and the calculated *p* < 0.05 (red) was considered significant. (**B**) The patient cohort in (**A)** was stratified into 2 groups according to the absence (−) or presence (+) of lymph node (LN) metastases, and the Spearman correlation (*r*) was determined as in (**A**). (**C**) The patient cohort in (**A)** was stratified into 4 groups according to the primary tumor stage, and the Spearman correlation (*r*) was determined as in (**A**). **(D**–**F**) The cohort of 40 SGC patients was stratified into groups as in (**A**–**C**), and the Spearman correlation (*r*) according to the expression of Fas in TIICs in the tumor periphery was determined.

We first investigated how the expression of Fas in TIICs in the center or periphery of the tumor correlated with disease severity. The data revealed that the frequency of tumors with Fas-expressing TIICs was notably higher in the tumor periphery than in its center (Figure 3). In the tumor center, Fas expression in TIICs correlated with none of the tested clinicopathological parameters (Figure 3A–C). No correlations were found with tumor grade or the presence of LN metastases in the tumor periphery (Figure 3D,E). However, tumor stage was found to negatively correlate with Fas expression in TIICs in the tumor periphery (Figure 3F). These data showed that the disease stage negatively impacted Fas-expressing TIICs in the periphery but not in the center of the tumors.

### 3.3. Fas Expression in Tumor Cells Positively Correlates with Tumor Stage in the Tumor Center

**Figure 4:**
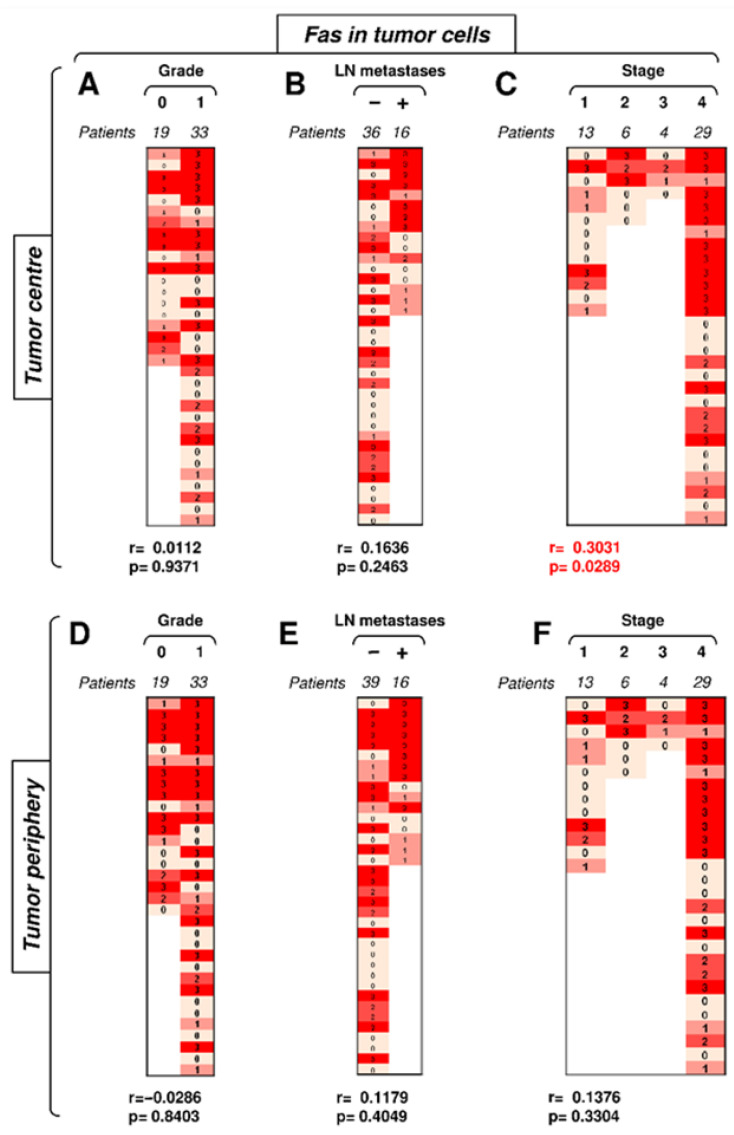
The expression of Fas in tumor cells in the tumor center correlates with tumor stage. (**A**) The cohort of 52 SGC patients was stratified into 2 groups according to tumor grade (grade 0 and grade 1), and the Spearman correlation (*r*) according to the expression of Fas in tumor cells in the tumor center was determined. The expression analyses were performed according to the scoring system described in the Materials and Methods section. The data are presented as a heat map with the indicated scores. The calculated *p* < 0.05 (red) was considered significant. (**B**) The patient cohort in (**A**) was stratified into 2 groups according to the absence (−) or presence (+) of lymph node (LN) metastases, and the Spearman correlation (*r*) was determined as in (**A**). (**C**) The patient cohort in (**A**) was stratified into 4 groups according to the primary tumor stage, and the Spearman correlation (*r*) was determined as in (**A**). (**D**–**F**) The cohort of 52 SGC patients was stratified into groups as in (**A**–**C**), and the Spearman correlation (*r*) according to the expression of Fas in tumor cells in the tumor periphery was determined.

We next analyzed whether Fas expression in tumor cells correlated with disease severity. The frequency and intensity of Fas expression in tumor cells were high in both tumor compartments (Figure 4). The compartmental analyses revealed no correlations of Fas expression in tumor cells in the tumor periphery (Figure 4D–F). Tumor grade and the presence of LN metastases did not correlate with Fas expression in tumor cells in the tumor center (Figure 4A,B). However, tumor stage showed a correlation in the tumor center (Figure 4C). These data showed that, contrary to TIICs, the disease stage positively impacted Fas-expressing tumor cells in the center but not periphery of the tumors.

### 3.4. FasL Expression in TIICs Is Not Impacted by Disease Severity or Compartmental Distribution in the Tumor

**Figure 5:**
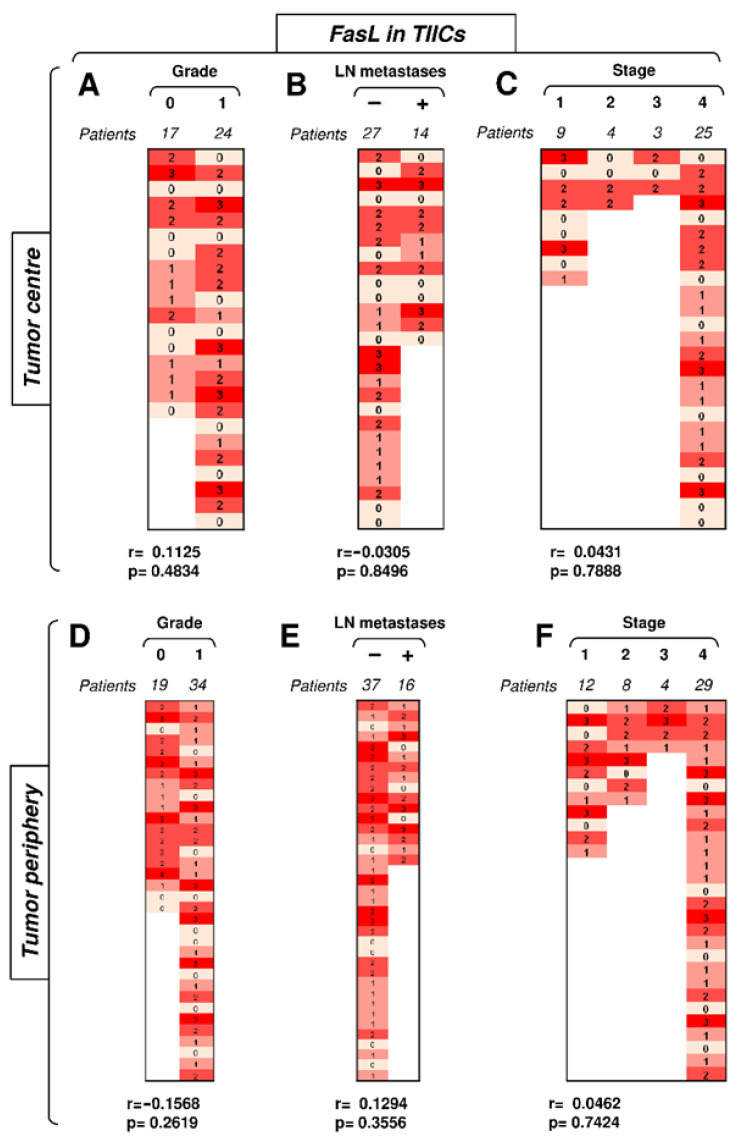
The expression of FasL in TIICs does not correlate with disease severity. (**A**) The cohort of 41 SGC patients was stratified into 2 groups according to tumor grade (grade 0 and grade 1), and the Spearman correlation (*r*) according to the expression of FasL in TIICs in the tumor center was determined. The expression analyses were performed according to the scoring system described in the Materials and Methods section. The data are presented as a heat map with the scores, and the calculated *p* < 0.05 (red) was considered significant. (**B**) The patient cohort in (**A**) was stratified into 2 groups according to the absence (−) or presence (+) of lymph node (LN) metastases, and the Spearman correlation (*r*) was determined as in (**A**). (**C**) The patient cohort in (**A**) was stratified into 4 groups according to the primary tumor stage, and the Spearman correlation (r) was determined as in (**A**). (**D**–**F**) The cohort of 53 SGC patients was stratified into groups as in (**A**–**C**), and the Spearman correlation (*r*) according to the expression of FasL in TIICs in the tumor periphery was determined.

The data showed that Fas expression in TIICs clustered the differences towards disease severity in the tumor periphery. Subsequent analyses, however, revealed that FasL expression in TIICs did not follow the same pattern. Despite its overall high frequency and intensity of expression in TIICs, no correlations of its expression with disease severity were found (Figure 5). Regardless of the compartmental distribution, tumor grade, the presence of LN metastases, and tumor stage did not correlate with FasL expression in TIICs (Figure 5). Therefore, unlike Fas, FasL expression in TIICs did not reflect disease severity.

### 3.5. FasL Expression in Tumor Cells Positively Correlates with Tumor Stage in the Tumor Periphery

**Figure 6:**
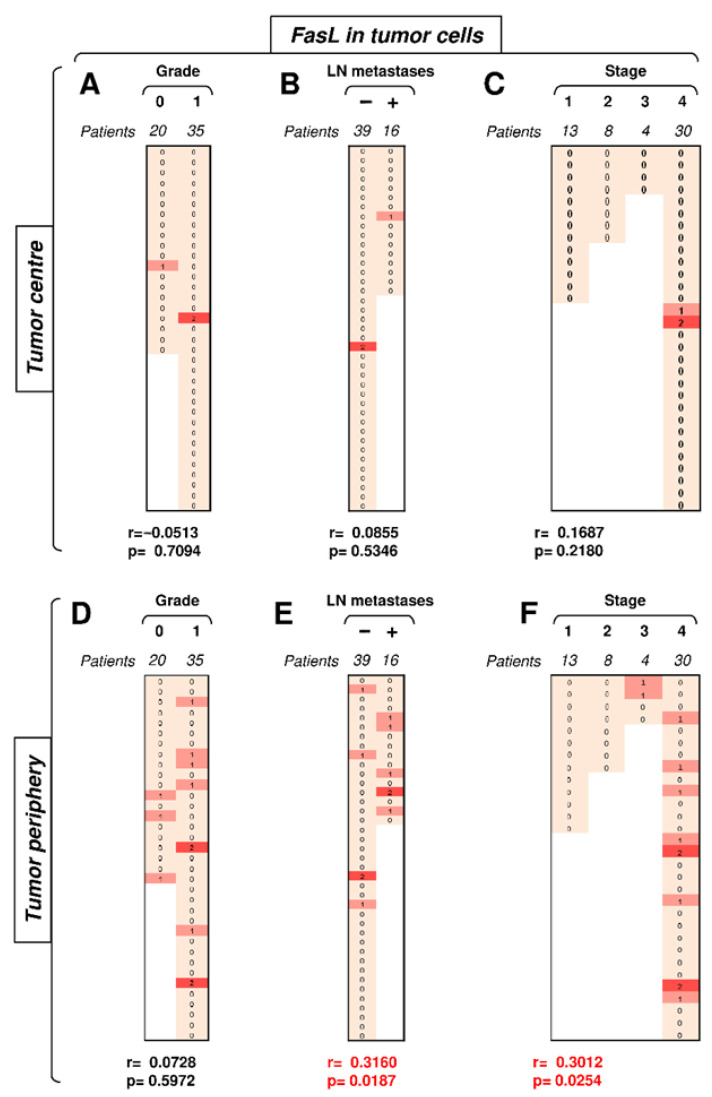
The expression of FasL in tumor cells in the tumor periphery correlates with tumor stage. (**A**) The cohort of 55 SGC patients was stratified into 2 groups according to tumor grade (grade 0 and grade 1), and the Spearman correlation (*r*) according to the expression of FasL in tumor cells in the tumor center was determined. The expression analyses were performed according to the scoring system described in the Materials and Methods section. The data are presented as a heat map with the indicated scores. The calculated *p* < 0.05 (red) was considered significant. (**B**) The patient cohort in (**A**) was stratified into 2 groups according to the absence (−) or presence (+) of lymph node (LN) metastases, and the Spearman correlation (*r*) was determined as in (**A**). (**C**) The patient cohort in (**A**) was stratified into 4 groups according to the primary tumor stage, and the Spearman correlation (*r*) was determined as in (**A)**. (**D**–**F**) The cohort of 52 SGC patients was stratified into groups as in (**A**–**C**), and the Spearman correlation (*r*) according to the expression of FasL in tumor cells in the tumor periphery was determined.

The data showed that Fas expression in tumor cells clustered the differences towards disease severity in the tumor center. In the subsequent analyses, we investigated FasL expression in these cells. Unlike Fas expression, FasL expression in tumor cells in the tumor center did not correlate with disease severity, and nearly all samples were negative for FasL in this compartment (Figure 6A–C). On the other hand, FasL expression in tumor cells in the tumor periphery was more frequent than that in the tumor center (Figure 6D–F). Importantly, however, FasL expression in this tumor compartment was already associated with disease severity. Whereas tumor grade showed only a tendency (Figure 6D, *p* = 0.0728), the presence of LN metastases or tumor stage already significantly correlated with FasL expression in tumor cells in the tumor periphery (Figure 6E–F). The most striking finding was the absence of peripheral FasL expression in stage 1 and 2 tumors but its frequent expression in stage 3 and 4 tumors (Figure 6F). These data showed that tumor cells of high-stage tumors could mobilize the Fas–FasL-mediated killing machinery in the tumor periphery of SGCs.

## 4. Discussion

This study showed that Fas and FasL in tumor cells and TIICs are differentially expressed in the tumor center and periphery. Our data revealed that Fas and FasL expression patterns in these cells within these two individual compartments formed signatures that reflected the severity of SGC and indicated a possible mechanism of SGC resistance to immunotherapy.

The binding of FasL to Fas serves as an apoptosis-inducing mechanism that immune cells can use to eliminate tumor cells [18]. However, tumor cells can also use this molecular mechanism to eliminate activated TIICs in tumors and promote tumor resistance to immunotherapy [15]. Our data showed that TIICs in SGCs expressed high levels of FasL in both tumor compartments, and these levels were much higher than the levels in tumor cells. This observation could, therefore, indicate that, upon FasL-induced apoptosis, TIICs have an advantage over tumor cells. However, FasL-induced apoptosis requires that Fas signaling in FasL-stimulated cells is intact and functional [29]. In tumor cells, this does not always occur because tumor cells can develop insensitivity to FasL-induced apoptosis [25,30,31,32]. Our data indeed suggest that this strategy could be employed by SGC cells because tumor cells expressed high levels of Fas in SGCs regardless of the presence of TIICs with high levels of FasL. Moreover, Fas expression in tumor cells in the tumor center was higher in the high-stage than in the low-stage tumors. This finding indicates that even though TIICs expressed much more FasL, this expression did not likely counteract the expanding Fas-expressing tumor cells.

The ineffective elimination of tumor cells via the Fas–FasL interaction could be a mechanism of immunoresistance in SGCs. However, our data also suggest that whereas immune cells are likely ineffective in the elimination of tumor cells via FasL [25,31,32], the tumor cell-mediated elimination of TIICs via FasL could be successful [27,33]. Two key findings from this study corroborated this suggestion. First, the tumor periphery of low-stage tumors was infiltrated with TIICs expressing high levels of Fas, but high-stage tumors had Fas expression in TIICs downregulated. Second, there was no expression of FasL in tumor cells in the periphery of low-stage tumors. However, its expression was found in their high-stage counterparts. This suggests that high-stage tumors could deplete Fas-expressing TIICs at the periphery of SGCs via FasL-induced apoptosis. The likelihood of this scenario is much higher because, compared to tumor cells, nontransformed cells are less likely to have a corrupt and nonfunctional Fas–FasL signaling axis, unless it is associated with a pathology [29,34,35,36]. In addition, FasL-induced apoptosis is key for the elimination of autoreactive lymphocytes under physiological conditions and is often utilized by tumors to promote their immunoresistance [27].

Immunotherapy for SGCs, including checkpoint inhibitors, has been unsuccessful [5]. It is difficult to define the mechanisms of tumor resistance in SGCs due to their extreme diversity. This diversity also translates into the diversity of the tumor immune microenvironment (TIME). TIME diversity in SGCs is well documented by observations that the most aggressive subtypes of SGCs largely differ in their TIME [37]. Our data, however, suggest that, regardless of the SGC subtype and TIME diversity, tumors may widely utilize the Fas–FasL signaling axis as a common mechanism to avoid elimination. On the one hand, this mechanism could ensure the resistance of tumor cells to FasL-induced apoptosis; on the other hand, these tumor cells may express FasL and induce apoptosis of TIICs at the periphery of high-stage tumors where FasL expression in tumor cells is enhanced. From this perspective, any immunotherapy design aimed at overcoming SGC resistance presumably based on this Fas–FasL-based mechanism should promote or use immune cells with enhanced resistance to FasL-induced apoptosis [38,39] and cytotoxicity, whose mode of action differs from FasL-induced apoptosis [40].

## 5. Conclusions

This study showed that Fas-expressing SGC tumor cells in the tumor center increase with increasing tumor stage, Fas-expressing tumor-infiltrating immune cells in the SGC tumor periphery decrease with increasing tumor stage, and FasL expression in SGC tumor cells in the tumor periphery correlates with tumor stage. These findings show that the Fas–FasL interplay in the periphery of SGC tumors represents a new checkpoint predictor for disease severity and immunotherapy response.

## Figures and Tables

**Table 1:** Histological subtypes (top panel) and the gender distribution of the histological subtypes (bottom panel) of the study cohort.

Mucoepidermoid Carcinoma	20.98%
Adenoid cystic carcinoma	17.74%
Acinic cell carcinoma	12.90%
Adenocarcinoma, not otherwise specified (NOS)	9.68%
Salivary duct carcinoma	9.68%
Undifferentiated carcinoma	6.45%
Carcinoma ex pleiomorphic adenoma	4.84%
Mammary analogue secretory carcinoma (MASC)	4.84%
Myoepithelial carcinoma	4.84%
Squamous cell carcinoma	3.23%
Adenosquamous carcinoma	1.61%
Carcinosarcoma	1.61%
Cribriform cystadenocarcinoma	1.61%
** 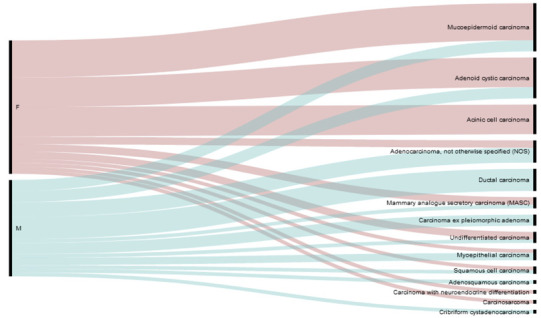 **	

**Table 2:** Study cohort: patients’ clinicopathological data.

SEX		AGE		GRADE		STAGE *		METASTASES	
Male	Female	50 year ≥ x	50 year ≤ x	Low (0)	High (1)	Stage (1 + 2)	Stage (3 + 4)	Yes (1)	No (0)
58.06%	42.94%	43.55%	56.45%	35.48%	64.52%	38.74%	59.68%	30.65%	69.35%

* In one patient (72 year), the primary tumor stage was not determined.

## Data Availability

Not applicable.
